# Sequence Analysis, Kinetic Constants, and Anion Inhibition Profile of the Nacrein-Like Protein (CgiNAP2X1) from the Pacific Oyster *Magallana gigas* (Ex-*Crassostrea gigas*)

**DOI:** 10.3390/md15090270

**Published:** 2017-08-28

**Authors:** Rosa Perfetto, Sonia Del Prete, Daniela Vullo, Giovanni Sansone, Carmela M. A. Barone, Mosè Rossi, Claudiu T. Supuran, Clemente Capasso

**Affiliations:** 1 Istituto di Bioscienze e Biorisorse, Consiglio Nazionale delle Ricerche (CNR), via Pietro Castellino 111, 80131 Napoli, Italy; Rosa.Perfetto@hotmail.it (R.P.); sonia.delprete@ibbr.cnr.it (S.D.P.); mose.rossi@ibbr.cnr.it (M.R.); 2 Sezione di Scienze Farmaceutiche and Laboratorio di Chimica Bioinorganica, Polo Scientifico, Dipartimento Neurofarba, Università degli Studi di Firenze, Via U. Schiff 6, Sesto Fiorentino, 50019 Florence, Italy; daniela.vullo@unifi.it; 3 Dipartimento di Biologia, Università degli Studi di Napoli, Federico II, via Cinthia 21, 80126 Napoli, Italy; giosanso@unina.it; 4 Dipartimento di Agraria, Università degli Studi di Napoli, Federico II, via Università 100, 80055 Portici (Napoli), Italy; cmbarone@unina.it

**Keywords:** nacrein-like protein, carbonic anhydrase, molluskan, signal peptide, kinetic constants, phylogeny, anion inhibitors

## Abstract

The carbonic anhydrase (CA, EC 4.2.1.1) superfamily of metalloenzymes catalyzes the hydration of carbon dioxide to bicarbonate and protons. The catalytically active form of these enzymes incorporates a metal hydroxide derivative, the formation of which is the rate-determining step of catalytic reaction, being affected by the transfer of a proton from a metal-coordinated water molecule to the environment. Here, we report the cloning, expression, and purification of a particular CA, i.e., nacrein-like protein encoded in the genome of the Pacific oyster *Magallana gigas* (previously known as *Crassostrea gigas*). Furthermore, the amino acid sequence, kinetic constants, and anion inhibition profile of the recombinant enzyme were investigated for the first time. The new protein, CgiNAP2X1, is highly effective as catalyst for the CO_2_ hydration reaction, based on the measured kinetic parameters, i.e., *k*_cat_ = 1.0 × 10^6^ s^−1^ and *k*_cat_/*K*_M_ = 1.2 × 10^8^ M^−1^·s^−1^. CgiNAP2X1 has a putative signal peptide, which probably allows an extracellular localization of the protein. The inhibition data demonstrated that the best anion inhibitors of CgiNAP2X1 were diethyldithiocarbamate, sulfamide, sulfamate, phenylboronic acid and phenylarsonic acid, which showed a micromolar affinity for this enzyme, with *K*_I_s in the range of 76–87 μM. These studies may add new information on the physiological role of the molluskan CAs in the biocalcification processes.

## 1. Introduction

The genome of Eubacteria, Archaea, and Eukarya encodes for a superfamily of metalloenzymes, known as carbonic anhydrases (CAs, EC 4.2.1.1). The CA superfamily includes seven known families: α-, β-, γ-, δ-, ζ-, η- and θ-CAs [1,2,3], which represent a fashionable example of convergent/divergent protein evolution. The main peculiarities of this superfamily can be summarized as follows: all the CA families aforementioned efficiently catalyze the hydration of carbon dioxide to bicarbonate and protons [4,5,6,7,8]; the catalytically active form of the enzyme is the metal hydroxide derivative [1,2,3]; the velocity of the catalytic reaction depends on the formation of a metal hydroxide species, which is formed by the transfer of a proton from the metal-coordinated water molecule from the enzyme active site to the surrounding solvent [2,3,4,6,7,8,9,10,11,12,13,14,15,16,17]. Taking into account the three-dimensional structure of the CA families, the seven genetic families are characterized by a different arrangement of the active site, with the metal ion playing an important part, as the apoenzymes are devoid of any catalytic activity. In fact, the metal ion in the active site is coordinated by three His residues in the α-, γ-, δ- and, probably, θ-classes; by one His, and two Cys residues in β- and ζ-CAs or by two His and one Gln residues in the η-class, with the fourth ligand being a water molecule/hydroxide ion acting as the nucleophile in the catalytic cycle of the enzyme [1,5,6,7,18,19]. The α-, β-, δ-, η- and, perhaps θ-CAs are characterized by a Zn(II) ion in the active site. γ-CAs are probably Fe(II) enzymes, although this family is active also with bound Zn(II) or Co(II) ions [20,21,22,23,24,25,26,27]. ζ-CAs are cambialistic enzymes, active both with Cd(II) or Zn(II) bound within the active site [28,29,30]. α-CAs are normally active as monomers or dimers; β-CAs are active only as dimers, tetramers, or octamers, whereas γ-CAs must be trimers for accomplishing their physiological function [21,22,25,31]. The crystal structure of ζ-CA showed three slightly different active sites on the same polypeptide chain. X-ray crystal structures of δ-, ζ-, η- and θ-CAs are not at all available. Intriguing, α- and η-CAs also catalyze the hydrolysis of esters/thioesters, while no esterase activity was detected for the other five CA families [1,32]. The CA gene families’ distribution is very variegated in living organisms investigated so far. In fact, CAs present in animals belong to α-class [33,34], plants and algae have α-, β-, γ-, δ- and θ-classes; fungi encode for α- and β-CAs; protozoa for α-, β- and/or η-CAs; bacteria for α-, β- and γ-CA classes [4,5,6,7,12,35,36]. In metazoans, the α-CAs are largely represented [37,38]. Moreover, in the mantle of the pearl oyster *Pinctada fucata* an enzyme involved in the calcium carbonate crystallization has been identified and indicated with the name of nacrein because it participates in the formation of the nacreous layer [39]. Nacrein is a matrix protein with both a CA domain at the N-terminus and Gly-Xaa-Asn repeats at the C-terminal part [39]. These proteins are conserved in bivalves and gastropods and have a molecular weight ranging from 50 kDa to 60 kDa [39,40]. Recently, a novel nacrein-like protein with a molecular weight of about 38 kDa was purified from the mantle of *P. fucata,* suggesting the existence of two kinds of CAs in the oyster [40]. Here, we cloned, expressed, and purified the recombinant nacrein-like protein (Accession number: XP_011428406.1) encoded by the genome of the Pacific oyster *Magallana gigas* (previously known as *Crassostrea gigas*). The protein is indicated here with the acronym CgiNAP2X. Furthermore, we investigated the amino acid sequence, kinetic constants, and anion inhibition profile of this enzyme, in order to add new information regarding the mineralization pathways of the molluskan organisms.

## 2. Results and Discussion

### 2.1. Amino Acid Sequence Analysis

**Figure 1:**
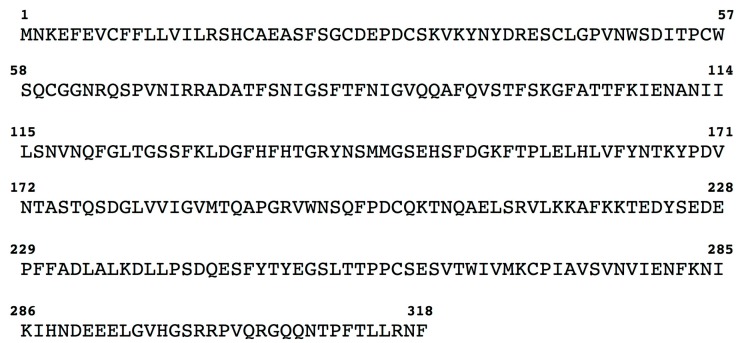
Amino acid sequence of the nacrein-like protein identified in the genome of *C. gigas*. The upper case letters are the amino acid residues.

By translated inspection of the *Crassostrea gigas* genome, we identified a gene encoding for a nacrein-like protein, which is indicated with the acronym CgiNAP2X1 in this article. The CgiNAP2X1 nucleotide sequence consists of an open reading frame encoding for a 318 amino acid polypeptide chain (Figure 1).

**Figure 2:**
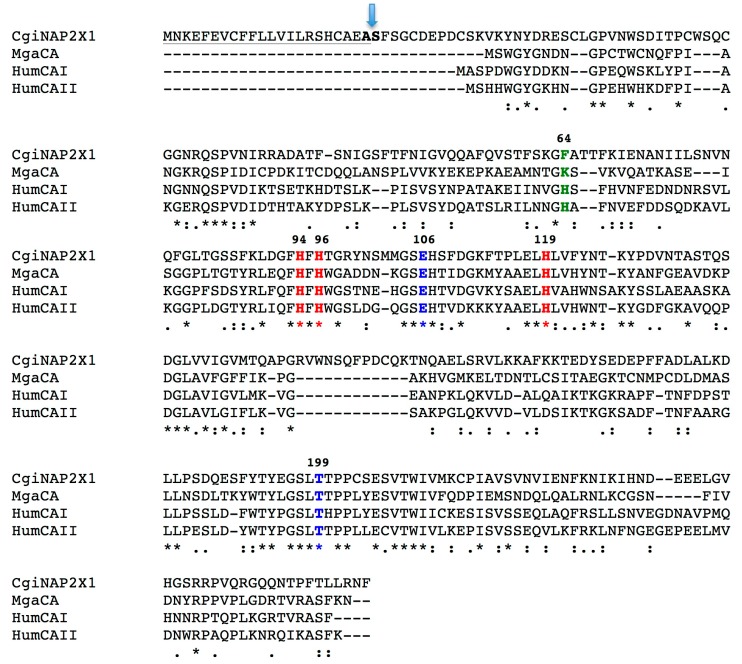
Alignment of the nacrein amino acid sequence identified in the genome of *Crassostrea gigas* (CgiNAP2X1, Accession number XP_011428406.1) with the α-CA from the bivalve *Mytilus galloprovincialis* (MgaCA, Accession number: ALF62133.1), and the two human α-CA isoforms (HumCAI, Accession number: NP001729 and HumCAII, Accession number: P00918). HumCAI numbering system was used. The zinc ligands (His94, 96, and 119 in red) and the gate-keeper residues (Glu106 and Thr199 in blue) are conserved in aligned sequences; while the proton shuttle residue (His64 in green) is preserved only in the human enzymes. The underlined residues represent the identified signal peptide, in bold the residues of the cleavage site, and the arrow is the position of the cleavage. The asterisk (*) indicates identity at all aligned positions; the symbol (:) relates to conserved substitutions, while (.) means that semi-conserved substitutions are observed. Multialignment was performed with the program MUSCLE, version 3.1.

To identify the typical features of the CgiNAP2X1 amino acid sequence, the protein was aligned with those of other α-CAs, such as the human isoforms HumCAI and HumCAII, and the molluskan MgaCA (Figure 2).

**Figure 3:**
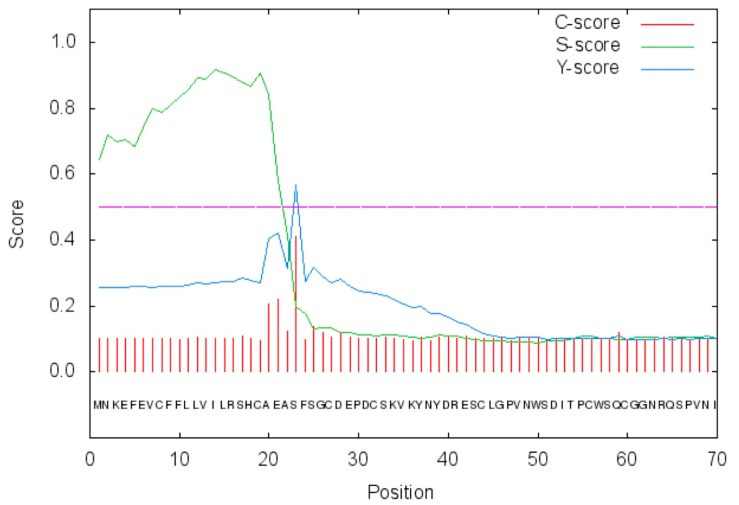
SignalP graphical output showing the three different scores C, S, and Y, for the first 70 positions in the CgiNAP2X1 amino acid sequence. Legend: *x*-axis, amino acid position; C-score, raw cleavage site score; S-score, signal peptide score; Y-score, combined cleavage site score.

It may be observed that similar to the other investigated α-CA members, CgiNAP2X1 has the conserved three His ligands which coordinate the Zn(II) ion crucial for catalysis (His94, 96, and 119, HumCAI numbering system), and the gate-keeping residues (Glu106 and Thr199) which orientate the substrate for catalysis, and are also involved in the binding of inhibitors. Interestingly, the proton shuttle residue (His64) is missed in CgiNAP2X1 and MgaCA (Figure 2). His64 assists the rate-determining step of the catalytic cycle, transferring a proton from the water coordinated to the Zn(II) ion to the environment, with formation of the zinc hydroxide, nucleophilic species of the enzyme. Moreover, from the amino acid alignment shown in Figure 2, we noted the presence of amino acids stretches at the N-terminus of the CgiNAP2X1 primary structure. This observation evoked our curiosity and a strong interest in the automated determination of a possible signal peptide in CgiNAP2X1. Here, we used SignalP version 4.1 (Center for Biological Sequence Analysis, Kongens Lyngby, Denmark), which is a program designed to discriminate between signal peptides and transmembrane regions of proteins [41]. The program is available as a web tool at http://www.cbs.dtu.dk/services/SignalP/. As shown in Figure 3, CgiNAP2X1 possessed a putative signal peptide of 22 amino acids at its N-terminus and a predicted cleavage site located between Ala22 and Ser23. A common feature of the human mitochondrial CAs (HumCAVA and HumCAVB), the salivary HumCAVI, all the bacterial α-CAs, and some bacterial β-CAs is the presence of an N-terminal signal peptide, which allows the inter-membrane, extracellular, or periplasmic localization of these CA-classes [9,32,42,43].

### 2.2. Phylogenetic Analysis

**Figure 4:**
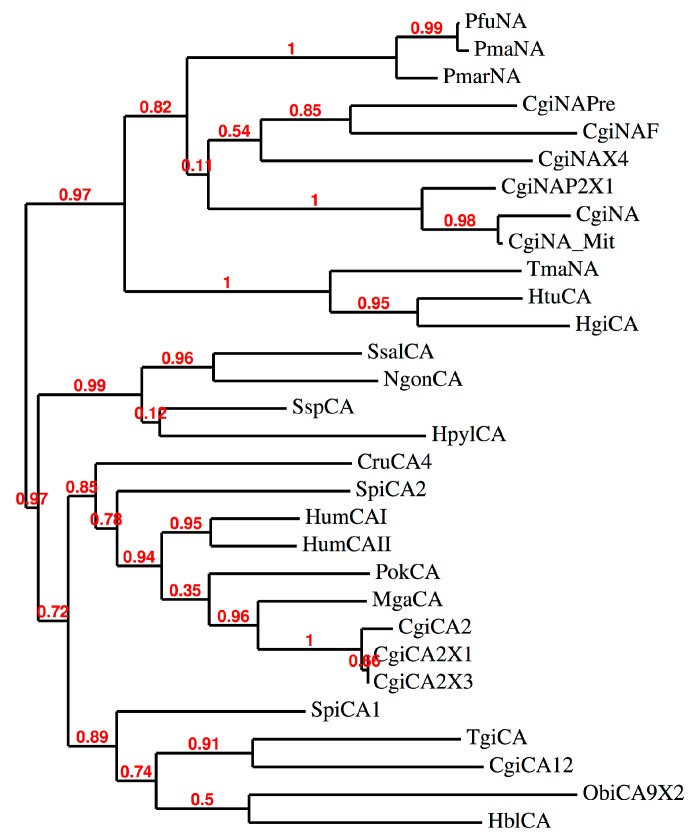
Phylogenetic analysis of CAs from various organisms. The analysis was obtained by using the amino acid sequences of nacrein and nacrein-like proteins identified in mollusks, and the α-CAs coming from molluskan, bacteria, and mammals. Bootstrap values of 100 replicates are reported at branch points. Legend: see Table 1 for sequence accession numbers, cryptonyms, and organisms considered.

We have constructed a phylogenetic tree in order to better investigate the evolutionary relationship of nacrein or nacrein-like sequences found in mollusks with that of other α-CAs coming from molluskan, bacteria, and mammals (Figure 4). The complete list of organisms, CA type, accession numbers and cryptonyms used in the phylogenetic analysis are indicated in Table 1.

The dendrogram shows that α-CAs and nacrein/nacrein-like proteins clustered in two distinct branches: one clade at the top of the Figure 4 containing all the nacreins, with the exception of the two α-CAs from *Haliotis tuberculata* (HtuCA) and *Haliotis gigantea* (HgiCA); the other clade (bottom of the Figure 4) included all the α-CAs considered in the present study. Interesting to note is the position of CgiNAP2X1. It is closely associated to the nacrein identified in the mitochondrial genome of *C. gigas*, which is characterized by a signal peptide similar to that found in CgiNAP2X1. When inspecting other molluskan nacrein or nacrein-like proteins deposited in the protein data bank and used for this phylogenetic analysis with SignalP, we found that nacreins, generally, are characterized by pre-sequences of 20 or more than 22 amino acid residues before the signal cleavage site (data not shown). These results could suggest that the identified nacrein-like protein, CgiNAP2X1, is a mitochondrial protein or a secretory protein, as happens for the human CAs isoforms Hum CAVA, HumCAVB, and HumaCAVI [9,32,42,43]. It is also possible to observe, that the clade of nacrein has arisen from an ancestral sequence, which led before to the bacterial α-CAs characterized by a signal peptide and then to the molluskan α-CA clade (Figure 4).

### 2.3. Production and Characterization of the Recombinant Enzyme (CgiNAP2X1) 

**Figure 5:**
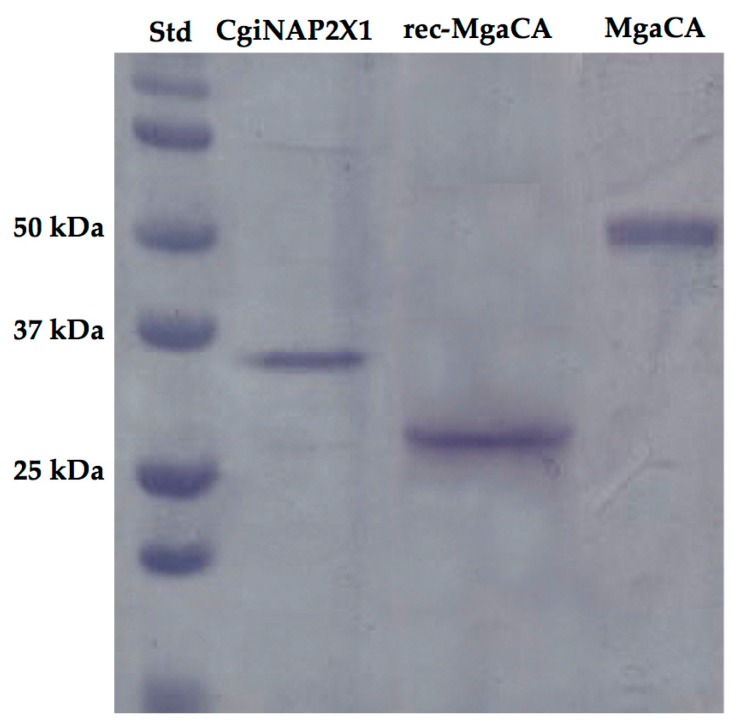
SDS-PAGE of the recombinant molluskan proteins (CgiNAP2X1 and rec-MgaCA) heterologously expressed in *Escherichia coli* and the native α-CA *M. galloprovincialis* (MgaCA). Lane STD, molecular markers, M.W. starting from the top: 150 kDa, 100 kDa, 75 kDa, 50 kDa, 37 kDa, 25 kDa, 20 kDa; Lane CgiNAP2X1, recombinant nacrein-like protein from *C. gigas*; rec-MgaCA, recombinant α-CA from *M. galloprovincialis*; Lane MgaCA; native α-CA purified from the mantle of *M. galloprovincialis*.

The recombinant CgiNAP2X1 was prepared by designing a synthetic gene lacking the peptide signal as described in “Materials and Methods” and heterologously expressed as a His-Tag fusion protein using the method reported earlier for several CAs [44]. The recombinant enzyme was recovered in the soluble fraction of the *Escherichia coli* ArcticExpress (DE3)RIL cells extract obtained after sonication and centrifugation. Using an affinity column (His-select HF Nickel Affinity Gel), CgiNAP2X1 was purified to an apparent homogeneity and the recovered amount of metalloenzyme was 2 mg. SDS-PAGE of the purified CgiNAP2X1 showed a band with an estimated molecular weight of about 36 kDa under reducing conditions (Figure 5). A subunit molecular mass of 37 kDa was calculated on the basis of the amino acid sequence translated from the gene.

**Figure 6:**
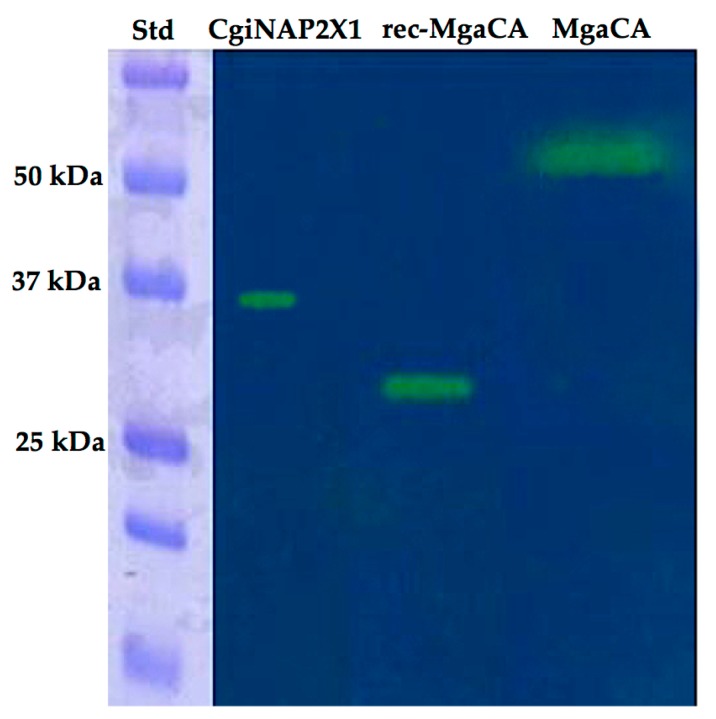
Protonogram showing the CgiNAP2X1, rec-MgaCA, and MgaCA activity on the gel. Protonography was run under denaturing but non-reducing conditions. The gel was loaded with 4 µg/well the aforementioned proteins and incubated for 20 for visualizing the hydratase activity bands (yellow color on the blue background). Lane STD, molecular markers, M.W. starting from the top: 75 kDa, 50 kDa, 37 kDa, 25 kDa, 20 kDa; Lane CgiNAP2X1, recombinant nacrein-like protein from *C. gigas*; rec-MgaCA, recombinant α-CA from *M. galloprovincialis*; Lane MgaCA; native α-CA purified from the mantle of *M. galloprovincialis*.

Interestingly, the native MgaCA purified from *Mytilus galloprovincialis* showed an estimated SDS-PAGE molecular weight of about 50 kDa, while the recombinant MgaCA (rec-MgaCA) had a molecular weight of about 30 kDa, which is closer to the molecular mass of CgiNAP2X1 (Figure 5). CgiNAP2X1 was also investigated by protonography, which is a technique based on monitoring the pH variation in the gel due to the CA-catalyzed conversion of CO_2_ to bicarbonate and protons [13,15,45]. The protonogram performed on CgiNAP2X1, rec-MgCA, and MgaCA showed a single hydratase activity band on the gel having a molecular weight of 36 kDa, 30 kDa, and 50 kDa, respectively (Figure 6), confirming the SDS-PAGE result. Moreover, since the dimer of the rec-MgaCA should have a M.W. of 60 kDa (10 kDa higher than the molecular mass of the native MgaCA), it is reasonable that the native MgaCA could be a multidomain protein with a CA domain at the N-terminus of the protein (M.W. = 30 KDa) and another domain of about 20 kDa at the C-terminus. This hypothesis is corroborated by the existence in the genome of the Japanese pearl oyster *Pinctada fucata* of a nacrein characterized by a CA domain at the N-terminus part of the protein [46].

### 2.4. Enzyme Kinetics

The data of Table 2 show that CgiNAP2X1 has a high catalytic activity for the physiological reaction of CO_2_ hydration to bicarbonate and protons, with the following kinetic parameters: *k*_cat_ of 1.0 × 10^6^ s^−1^ and *k*_cat_/*K*_M_ of 1.2 × 10^8^ M^−1^·s^−1^ (at 20 °C and pH of 7.5), being thus 1.4 times less active than the human isoform HumCAII, which, together with SazCA (α-CA from the thermophilic bacteria *Sulfurihydrogenibium azorense* with a *k*_cat_ of 4.4 × 10^6^ s^−1^) is of one of the best catalysts known in nature. Moreover, CgiNAP2X1 is one order of magnitude more effective as a catalyst for the physiological reaction compared to a similar molluskan recombinant enzyme and the native enzyme (MgaCA) and with respect to the slow human HumCAI and the other α-CAs identified in species of corals (SpiCA1 and SpiCA2 from *Stylophora pistillata*; CruCA4 from *Corallium rubrum*) (Table 2). Interestingly, the sulfonamide in clinical use, acetazolamide (5-acetamido-1,3,4-thiadiazole-2-sulfonamide), is an excellent inhibitor of HumCAII, SpiCA1, and SpiCA2 (*K*_I_s = 12–74 nM), while it was less effective towards CgiNAP2X1, HumCAI, CruCA4, and MgaCA (native and recombinant) with inhibition constants in the range of 250–450 nM. These activity data cannot be easily rationalized taking into consideration the amino acid sequence of CgiNAP2X1, which has been aligned with the α-CA from the bivalve *M. galloprovincialis*, and the two human α-CA isoforms (Figure 2). In fact, in the amino acid sequence of CgiNAP2X1, the proton shuttle residue His64 is missing, which is normally responsible for the velocity of the catalytic cycle (see paragraph 2.1 and Figure 2). Probably, the *k*_cat_ of 1.0 × 10^6^ s^−1^ showed by CgiNAP2X1 is attributed to other amino acid sequence features, such as the presence of an amino acid insertion positioned at the half of the primary structure (Figure 2), which could increase the flexibility of the molecules determining a higher *k*_cat_. Of course, further studies are needed to demonstrate this observation.

### 2.5. Anion Inhibition Profile 

As inorganic anions represent a well-known class of CA inhibitors (CAIs) due to their affinity for metal ions in solution or when bound within metalloenzyme active sites, we investigated a rather large number of such species for their interaction with CgiNAP2X1 (Table 3). The interest in this class of CAIs is due to the fact that *Crassostrea gigas* lives in the marine environment, which may contain various concentrations of salts and, thus, a rather high amount of inorganic anions. Data of Table 3, in which the inhibition of the human enzymes HumCAI and II is reported for comparison reasons, show the following:

1. The anions with the smallest propensity to coordinate metal ions, perchlorate and tetrafluoroborate, and inhibitors such as fluorosulfonate, iminodisulfonate, perrhuthenate, perrhenate, bisulfite, divanadate, tellurate, azide, and cyanide were not inhibitors of CgiNAP2X1 up to concentrations of 100 mM. With the exception of perchlorate and tetrafluoroborate, a different profile was shown for the human isoforms hCA I and II (Table 3).
2. Several other anions; fluoride, chloride, bromide, iodide, cyanate, thiocyanate, bicarbonate, carbonate, nitrate, nitrite, hydrogen sulfite, stannate, selenate, peroxydisulfate, tetraborate, selenocyanate, trithiocarbonate, and trifluorometansolfonato were also weak inhibitors of CgiNAP2X1, with inhibition constants in the range of 3.1–20.7 mM (Table 3). Interesting to note is that most of these inhibitors were micro and/or submicromolar inhibitors for the human enzymes. It should be noted that the weak affinity of CgiNAP2X1 for bicarbonate and carbonate might be a positive feature for the physiological role of this enzyme in the deposition of the molluskan shell, since the enzyme is not appreciably inhibited in the presence of rather high concentrations of these two anions. Moreover, for the same reason, the enzyme could have also a potential biotechnological application in the CO_2_ capture processes.
3. The best anion inhibitors of CgiNAP2X1 were diethyldithiocarbamate, sulfamide, sulfamate, phenylboronic acid, and phenylarsonic acid, which showed a micromolar affinity for this enzyme, with *K*_I_s in the range of 76–87 μM (Table 3). Diethyldithiocarbamate was recently shown to lead to highly potent inhibitors directed against many α- or β-CAs, and some X-ray crystal structures also revealed their binding mode, which is achieved by coordination to the metal ion via a negatively charged sulfur atom [47,48,49,50,51]. It is conceivable that a similar binding mode is achieved also within the CgiNAP2X1 active site. Sulfamide and sulfamate also coordinate (via an ionized nitrogen atom) to the Zn(II) ion within the hCA II active site, and probably the same inhibition mechanism is valid against the oyster enzyme [47,48,49,50,51].
4. Most of these anions are weak, millimolar CgiNAP2X1 inhibitors probably because the enzyme is in contact with sea water, rich in many inorganic anions (chloride, sulfate, etc.) which may interfere with the enzyme’s activity. Similar behavior was observed earlier for other marine organism CAs [37].

## 3. Materials and Methods

### 3.1. Gene Identification, Synthesis, and Cloning

The identification of the gene encoding for the *Crassostrea gigas* nacrein-like protein (CgiNAP2X1) was performed following the strategy reported below. Briefly, the CgiNAP2X1 gene of *C. gigas* with the accession number XP_011428406.1 was identified running the “Protein BLAST” program and using as query sequence the amino acid sequence of the α-CA purified previously from the mantle of the Mediterranean mussel, *Mytilus galloprovincialis* [37]. The GeneArt Company (Thermo Fisher Scientific, Milan, Italy), specialized in gene synthesis, designed the synthetic CgiNAP2X1 gene without the peptide signal and having at the 5′ end four base-pair sequences (CACC) necessary for the directional cloning in the pMK-T vector (subcloning vector, Thermo Fisher Scientific, Milan, Italy). The CgiNAP2X1 was subsequently cloned into the expression vector pET100/D-TOPO (Invitrogen, Palo Alto, CA, USA), creating the plasmid pET100D-Topo/CgiNAP2X1. In order to confirm the gene integrity and that no errors occurred at the ligation sites, the vector containing the fragment was subjected to bidirectional automated sequencing.

### 3.2. Protein Expression and Purification

*Escherichia coli* ArcticExpress (DE3)RIL competent cells were transformed with pET100D-Topo/CgiNAP2X1, grown at 20 °C, and induced with 1 mM IPTG. ZnSO_4_ was added after 30 min and after additional growth for 10 h, cells were harvested and disrupted by sonication at 4 °C in 20 mM buffer phosphate, pH 8.0. Following sonication, the sample was centrifuged at 1200 *g* at 4 °C for 30 min. The supernatant was dialyzed at 4 °C against 0.02 M phosphate buffer (pH 8.0) containing 0.01 M imidazole and loaded onto a His-select HF Nickel affinity column (GE Healthcare, dimension: 1.0 × 10 cm). The column was equilibrated with 0.02 M phosphate buffer (pH 8.0) containing 0.01 M imidazole and 0.5 M KCl at a flow rate of 1.0 mL/min. The recombinant CgiNAP2X1 was eluted from the column with 0.02 M phosphate buffer (pH 8.0) containing 0.5 M KCl and 0.3 M imidazole at a flow rate of 1.0 mL/min. Active fractions (0.5 mL) were collected and combined for a total volume of 2.5 mL. Subsequently, they were dialyzed, concentrated, and analyzed by SDS-PAGE. At this stage of purification the enzyme was at least 95% pure and the obtained recovery was of 2.0 mg.

### 3.3. SDS-PAGE and Protonography

Sodium dodecyl sulfate (SDS)-polyacrylamide gel electrophoresis (SDS-PAGE) was performed as described by Laemmli using 12% gels [53]. For the protonography, the samples were mixed in a loading buffer without 2-mercaptoethanol and they were not boiled, in order to avoid protein denaturation. The gel was run at 150 V until the dye front ran off the gel. Following the electrophoresis, the gel was subject to protonography to detect the hydratase activity of the samples as described by Clemente Capasso and coworkers [45,54,55].

### 3.4. Amino Acid Sequence and Phylogenetic Analysis

Multialignment of amino acid sequences was performed using the program MUSCLE 3.1 (MUltiple Sequence Comparison by Log-Expectation), a new computer program for creating multiple alignments of protein sequence [56]. The dendrogram was constructed using the program PhyML 3.0 searching for the tree with the highest probability [57].

### 3.5. Enzyme Kinetics

An Applied Photophysics stopped-flow instrument has been used for assaying the CA catalyzed CO_2_ hydration activity [52]. Phenol red (at a concentration of 0.2 mM) has been used as indicator, working at the absorbance maximum of 557 nm, with 20 mM TRIS (pH 8.3) as buffer, and 20 mM NaClO_4_ (for maintaining constant the ionic strength), following the initial rates of the CA-catalyzed CO_2_ hydration reaction for a period of 10–100 s. The CO_2_ concentrations ranged from 1.7 mM to 17 mM for the determination of the kinetic parameters (by Lineweaver–Burk plots) and inhibition constants. For each inhibitor at least six traces of the initial 5–10% of the reaction have been used for determining the initial velocity. The uncatalyzed rates were determined in the same manner and subtracted from the total observed rates. Stock solutions of inhibitor (10–100 mM) were prepared in distilled-deionized water and dilutions up to 0.01 mM were done thereafter with the assay buffer. Inhibitor and enzyme solutions were preincubated together for 15 min at room temperature prior to assay, in order to allow for the formation of the E-I complex or for the eventual active site mediated hydrolysis of the inhibitor. The inhibition constants were obtained by non-linear least-squares methods using PRISM 3 and the Cheng-Prusoff equation, as reported earlier [58,59,60], and represent the mean from at least three different determinations. All CA isoforms were recombinant ones obtained in-house. All salts/small molecules were of the highest purity available, from Sigma-Aldrich (Milan, Italy).

## 4. Conclusions

In mollusks, CA activity has been detected in mantle, gill, muscle, and blood, and participates in the process of acid-base regulation, calcification, and mineralization, providing inorganic carbon at the site of calcification and/or determining the precipitation of calcium carbonate [61,62,63,64,65,66,67,68,69,70,71,72]. Recently, we characterized and determined the kinetic constants of the CA purified from the mantle tissue of the bivalve Mediterranean mussel, *Mytilus galloprovincialis*. The SDS-PAGE, protonography, and kinetic analyses give strength to our hypothesis that the native MgCA is probably a multidomain protein with a single CA domain at the N-terminus. The present work is the first study on the kinetic constants and the anion inhibition profile of the recombinant nacrein-like protein from *M. gigas*. The enzyme is highly effective at catalysis based on the measured kinetic parameters for the CO_2_ hydration reaction (*k*_cat_ of 1.0 × 10^6^ s^−1^ and a *k*_cat_/*K*_M_ of 1.2 × 10^8^ M^−1^ s^–1^). The CgiNAP2X1 amino acid sequence is characterized by a putative signal peptide at the N-terminus part of the protein, which probably allows an extracellular localization of the protein. The inhibition data demonstrated that the best anion inhibitors of CgiNAP2X1 were diethyldithiocarbamate, sulfamide, sulfamate, phenylboronic acid, and phenylarsonic acid, which showed a micromolar affinity for this enzyme, with *K*_I_s in the range of 76–87 µM. This study may add new information on metabolic pathways of the biocalcification processes, which involves CAs, nacreins, and nacrein-like proteins. Moreover, this enzyme could also be potentially used in the biomimetic capture of the CO_2_ due to its high *k*_cat_ and the low inhibition towards bicarbonate and carbonate. It is important to note that in many other marine organisms, such as corals [69,70,71,72] and sponges [73,74], CAs are crucial in biomineralization processes [69,70,71,72,73,74], and understanding the biochemical mechanisms by which these organisms build their skeletons may have important environmental and even biotechnological applications. As the mollusk CAs are less investigated, we consider this article an interesting opportunity for a deeper understanding of these proteins in this type of marine organism.

## Figures and Tables

**Table 1:** Cryptonyms, enzyme types, organisms, and accession numbers of the amino acid sequences used in the phylogenetic analysis.

Acronym	Enzyme Type	Organism	Accession Number
PfuNA	Nacrein	*Pinctada fucata*	BAA11940.1
PmaNA	Nacrein-like	*Pinctada maxima*	BAF42330.1
PmarNA	Nacrein	*Pinctada margaritifera*	AEC03970.1
CgiNAPre	Nacrein precursor	*Crassostrea gigas*	NP_001292235.1
CgiNAF	Nacrein isoform F	*Crassostrea gigas*	EKC37961.1
CgiNAX4	Nacrein isoform X4	*Crassostrea gigas*	XP_011426380.1
CgiNAP2X1	Nacrein-like isoform P2X1	*Crassostrea gigas*	XP_011428406.1
CgiNA	Nacrein-like	*Crassostrea gigas*	XP_011428408.1
CgiNA_Mit	Mitochondrial nacrein	*Crassostrea gigas*	EKC36446.1
TmaNA	Nacrein	*Turbo marmoratus*	BAB91157.1
HtuCA	α-CA	*Haliotis tuberculata*	AEL22200.1
HgiCA	α-CA	*Haliotis gigantea*	BAH58350.1
SsalCA	α-CA	*Streptococcus salivarius]*	WP_002888224.1
NgonCA	α-CA	*Neisseria gonorrhoeae*	WP_003688976.1
SspCA	α-CA	*Sulfurihydrogenibium yellowstonense*	WP_012459296.1
HpylCA	α-CA	*Helicobacter pylori*	WP_010882609.1
CruCA4	α-CA isoform 4	*Corallium rubrum*	KU557746.1
SpiCA2	α-CA isoform 2	*Stylophora pistillata*	ACE95141.1
HumCAI	α-CA isoform 1	*Homo sapiens*	NP_001729.1
HumCAII	α-CA isoform 2	*Homo sapiens*	AAH11949.1
PokCA	α-CA	*Phreagena okutanii*	BAO01209.1
MgaCA	α-CA	*Mytilus galloprovincialis*	ALF62133.1
CgiCA2	α-CA isoform 2	*Crassostrea gigas*	EKC31880.1
CgiCA2X1	α-CA isoform 2X1	*Crassostrea gigas*	XP_011447892.1
CgiCA2X3	α-CA isoform 2X3	*Crassostrea gigas*	XP_011447895.1
SpCA1	α-CA isoform 1	*Stylophora pistillata*	ACA53457.1
TgiCA	α-CA	*Tridacna gigas*	AAX16122.1
CgiCA12	α-CA isoform 12	*Crassostrea gigas*	EKC34762.1
ObiCA9X	α-CA isoform 9X	*Octopus bimaculoides*	XP_014786584.1
HblCA	α-CA	*Heterololigo bleekeri*	BAL42244.1

**Table 2:** Comparison of the kinetic parameters for the CO_2_ hydration reaction catalyzed by CgiNAP2X1 (nacrein) and the α-CAs from *Homo sapiens* (the two isoforms HumCAI and HumCAII), *Stylophora pistillata* (isoforms SpiCA1 and SpiCA2), *Corallium rubrum* (isoform CruCA4), *Mytilus galloprovincialis* (the native enzyme, MgCA; and the recombinant form, rec-MgaCA). Acetazolamide (AAZ) inhibition data are also shown.

Enzyme	Organism	Class	*k*_cat_^1^ (s^−1^)	*k*_cat_/*K*_M_^1^ (M^−1^ × s^−1^)	*K*_I_s (acetazolamide) ^1^ (nM)
HumCAI	*Homo sapiens*	α	2.0 × 10^5^	5.0 × 10^7^	250
HumCAII	*Homo sapiens*	α	1.4 × 10^6^	1.5 × 10^8^	12
CgiNAP2X1	*Crassotrea gigas*	Nacrein-like	1.0 × 10^6^	1.2 × 10^8^	495
SpiCA1	*Stylophora pistillata*	α	3.1 × 10^5^	4.6 × 10^7^	16
SpiCA2	*Stylophora pistillata*	α	5.6 × 10^5^	8.3 × 10^7^	74
CruCA4	*Corallium rubrum*	α	2.4 × 10^5^	5.2 × 10^7^	450
MgaCA	*Mytilus galloprovincialis*	α	4.1 × 10^5^	3.6 × 10^7^	380
rec-MgaCA	*Mytilus galloprovincialis*	α	4.2 × 10^5^	3.5 × 10^7^	361

^1^ Errors in the range of ±5% of the reported data from three different assays.

**Table 3:** Inhibition constants of anion inhibitors against α-CAs from mammals (hCA I, and II, human isoforms), and the nacrein-like enzyme CgiNAP2X1 for the CO_2_ hydration reaction, at 20 °C and pH 7.5 [52].

Inhibitor ^1^	*K*_I_s [mM] ^2^		
	hCA I	hCA II	CgiNAP2X1
F^−^	>100	>100	12.1
Cl^−^	6	>100	10.8
Br^−^	4	63	16.6
I^−^	0.3	26	8.86
CNO^−^	0.0007	0.03	3.11
SCN^−^	0.2	1.60	5.88
CN^−^	0.0005	0.02	>100
N_3_^−^	0.0012	1.51	>100
HCO_3_^−^	12	85	1.98
CO_3_^2−^	15	73	6.62
NO_3_^−^	7	35	10.3
NO_2_^−^	8.4	63	7.14
HS^−^	0.0006	0.04	8.19
HSO_3_^−^	18	89	>100
SnO_3_^2−^	0.57	0.83	3.47
SeO_4_^2−^	>100	>100	8.74
TeO_4_^2−^	0.66	0.92	>100
OsO_5_^2−^	0.92	0.95	>100
P_2_O_7_^4−^	25.8	48.5	9.47
V_2_O_7_^4−^	0.54	0.57	>100
B_4_O_7_^2−^	0.64	0.95	8.85
ReO_4_^−^	0.11	0.75	>100
RuO_4_^−^	0.101	0.69	>100
S_2_O_8_^2−^	0.107	0.084	20.7
SeCN^−^	0.085	0.086	8.21
CS_3_^2−^	0.0087	0.0088	31.7
Et_2_NCS_2_^−^	0.00079	0.0031	0.76
CF_3_SO_3_^−^	>100	>100	8.68
PF_6_^−^	>100	>100	>100
SO_4_^2−^	63	>100	14.5
ClO_4_^−^	>100	>100	>100
BF_4_^−^	>100	>100	>100
FSO_3_^−^	0.79	0.46	>100
NH(SO_3_)_2_^2−^	0.31	0.76	>100
H_2_NSO_2_NH_2_	0.31	1.13	0.076
H_2_NSO_3_H	0.021	0.39	0.073
Ph-B(OH)_2_	58.6	23.1	0.072
Ph-AsO_3_H_2_	31.7	49.2	0.087

^1^ As sodium salt, except sulfamide, phenylboronic acid, and phenylarsonic acid; ^2^ Errors were in the range of 3–5% of the reported values, from three different assays, by a CO_2_ hydratase assay method [52].
